# The DNA Phosphorothioation Restriction-Modification System Influences the Antimicrobial Resistance of Pathogenic Bacteria

**DOI:** 10.1128/spectrum.03509-22

**Published:** 2023-01-04

**Authors:** Congrui Xu, Jing Rao, Yuqing Xie, Jiajun Lu, Zhiqiang Li, Changjiang Dong, Lianrong Wang, Jinghong Jiang, Chao Chen, Shi Chen

**Affiliations:** a Brain Center, Department of Neurosurgery, Key Laboratory of Combinatorial Biosynthesis and Drug Discovery Ministry of Education, Zhongnan Hospital of Wuhan University, School of Pharmaceutical Sciences, Wuhan University, Wuhan, China; b Department of Obstetrics & Gynecology, Zhongnan Hospital of Wuhan University, Wuhan University, Wuhan, China; c Information Engineering Institute, Wuchang Institute of Technology, Wuhan, China; d Department of Burn and Plastic Surgery, Shenzhen Institute of Translational Medicine, Health Science Center, Shenzhen Second People's Hospital, The First Affiliated Hospital of Shenzhen University, Shenzhen, China; South China Sea Institute of Oceanology

**Keywords:** DNA phosphorothioation, restriction-modification, antimicrobial resistance, horizontal gene transfer

## Abstract

Bacterial defense barriers, such as DNA methylation-associated restriction-modification (R-M) and the CRISPR-Cas system, play an important role in bacterial antimicrobial resistance (AMR). Recently, a novel R-M system based on DNA phosphorothioate (PT) modification has been shown to be widespread in the kingdom of *Bacteria* as well as *Archaea*. However, the potential role of the PT R-M system in bacterial AMR remains unclear. In this study, we explored the role of PT R-Ms in AMR with a series of common clinical pathogenic bacteria. By analyzing the distribution of AMR genes related to mobile genetic elements (MGEs), it was shown that the presence of PT R-M effectively reduced the distribution of horizontal gene transfer (HGT)-derived AMR genes in the genome, even in the bacteria that did not tend to acquire AMR genes by HGT. In addition, unique gene variation analysis based on pangenome analysis and MGE prediction revealed that the presence of PT R-M could suppress HGT frequency. Thus, this is the first report showing that the PT R-M system has the potential to repress HGT-derived AMR gene acquisition by reducing the HGT frequency.

**IMPORTANCE** In this study, we demonstrated the effect of DNA PT modification-based R-M systems on horizontal gene transfer of AMR genes in pathogenic bacteria. We show that there is no apparent association between the genetic background of the strains harboring PT R-Ms and the number of AMR genes or the kinds of gene families. The strains equipped with PT R-M harbor fewer plasmid-derived, prophage-derived, or integrating mobile genetic element (iMGE)-related AMR genes and have a lower HGT frequency, but the degree of inhibition varies among different bacteria. In addition, compared with Salmonella enterica and Escherichia coli, Klebsiella pneumoniae prefers to acquire MGE-derived AMR genes, and there is no coevolution between PT R-M clusters and bacterial core genes.

## INTRODUCTION

With the continued abuse of various antibiotics in clinical settings, an increasing number of pathogenic bacteria have developed noticeable antimicrobial resistance (AMR), and many bacteria have even adapted multiple molecular mechanisms to resist different types of antibiotics. In early 2017, the World Health Organization released a list of pathogens for which research and development for antibiotic treatment need to be prioritized, including a variety of clinically common opportunistic pathogens, such as Acinetobacter baumannii, Pseudomonas aeruginosa, Klebsiella pneumoniae, and Salmonella enterica (1). The mechanisms by which bacteria resist antibiotics are divided into three main categories, (i) hydrolase activity-targeting antibiotics; (ii) prevention of drug entry into cells, and (iii) mutation of antibiotic target sites (2). The genes associated with drug resistance in bacterial genomes are called AMR genes, and there are currently a variety of databases or software that can be used to predict and analyze these AMR genes (3–5). Since AMR bacteria have become a worldwide problem, an increasing number of studies have focused on the transmission of AMR genes. Horizontal gene transfer (HGT) greatly contributes to the rapid spread of drug resistance (6). Mobile genetic elements (MGEs), including plasmids, transposons, gene cassettes/integrons, insertion sequences, and integrative conjugative elements, provide a source of transmissible resistance genes for this process (7). Furthermore, recent studies have revealed the role of bacteriophages in AMR gene transmission, as the AMR gene could be encoded in the genome of prophages or packaged in phage particles (8, 9).

To maintain the stability of their own genetic material, bacteria have evolved diverse defense systems, including the DNA methylation-related (Me) R-M system and CRISPR-Cas system, against the invasion of genetic parasites. Studies have shown that Me R-M systems are involved in processes that influence bacterial AMR by affecting the uptake of exogenous AMR genes. A study of the Me R-M system in Enterococcus faecalis strain OG1RF revealed that this strain has few MGEs in its genome and there is global 5-methylcytosine (m5C) methylation at the 5′-GCWGC-3′ motifs. This type II R-M system provides a modest but important defense against the model pheromone-responsive plasmid pCF10, the absence of which affects the electrotransformability of OG1RF and the conjugative transfer of AMR plasmids (10, 11).

As another important defense system of bacteria, CRISPR-Cas can affect the acquisition of exogenous AMR genes by bacteria as well. It was found that among clinical isolates of K. pneumoniae, strains with the type I-E CRISPR-Cas system were less resistant to multiple antibiotics than those without the CRISPR-Cas system (12). In Bacillus cereus, the predicted levels of transposases, plasmid replication proteins, and prophage elements in strains with an intact CRISPR-Cas system were significantly lower than those in strains with an incomplete CRISPR-Cas system or those lacking the CRISPR-Cas system (13). This shows that the CRISPR-Cas system has an inhibitory effect on HGT.

Recently, a novel R-M system involving phosphorothioate (PT) modification in the DNA backbone has been revealed (14–16). The modification module, consisting of DndABCDE five proteins, transferred sulfur atoms from l-cysteine to swap the nonbridging oxygen in the DNA phosphodiester bond in a sequence-specific manner (17). In sharp contrast to DNA methylation, PTs exhibit cell-to-cell heterogeneity (18, 19). Interestingly, the restriction module composed of DndFGH could adapt to this unusual modification pattern and form an unusual R-M system (16). In addition to participating in the defense barrier, PT modification was demonstrated to contribute to the overall cellular redox state and have a potential role in epigenetic regulation (20–23).

We speculate that this PT-based R-M system may also have potential effects on bacterial AMR. Through a series of bioinformatics analyses of strain populations of several common clinical pathogens, we found that, similar to the classic Me R-M system or CRISPR-Cas system, the PT R-M system reduces the horizontal transfer frequency of MGE-related AMR genes, thereby reducing the distribution of AMR genes in the bacterial genome. However, the degree of inhibition by PT R-Ms on AMR gene distribution varied among bacteria due to inconsistent methods of AMR gene acquisition.

## RESULTS

### Impacts of the PT R-M system on the distribution of AMR genes in K. pneumoniae, E. coli, and S. enterica.

To explore whether the presence of the PT R-M system has an impact on the AMR gene distribution, BLAST was performed based on the data from the NCBI Assembly database according to the WHO priority pathogens list for research and development (R&D) of new antibiotics (1). First, we analyzed the distribution of the PT R-M system in pathogenic bacteria involved in the list to screen suitable candidate species for efficient statistical analysis. We downloaded all the assembly RefSeq files that provide only a single record for each natural biological molecule, ruling out the possibility of repeating statistics on the same sample (24), from the NCBI database and built a sample pool for local screening of strains with the PT R-M gene cluster. We found 56, 1,206, and 425 strains harboring PT R-M clusters out of 8,873 Klebsiella pneumoniae, 22,747 Escherichia coli, and 11,354 Salmonella enterica strains, respectively, compared to only 3 strains harboring PT R-M clusters out of 3,970 Acinetobacter baumannii strains (see Tables S1 and S2 in the supplemental material). In contrast, no *dndBCDE-FGH* was found in Pseudomonas aeruginosa (5,006 strains), Enterococcus faecium (2,009 strains), Staphylococcus aureus (10,741 strains), or Helicobacter pylori (1,672 strains). Therefore, K. pneumoniae, E. coli, and S. enterica were selected for further analysis.

According to the data given above, 385 strains of K. pneumoniae, 352 strains of E. coli, and 875 strains of S. enterica, including 56, 150, and 425 strains harboring PT R-M clusters, respectively, were randomly selected, and the quality of these genome files was checked. For K. pneumoniae, E. coli, and S. enterica, the number of contigs in 99.48% (383 out of 385), 98.01% (345 out of 352), and 98.86% (865 out of 875) of selected samples was less than 500, ranging from 1 to 394, 1 to 491, and 1 to 473, respectively, and the average length of their contigs ranged from 14,434.8 bp to 5,568,802 bp, 10,509 bp to 5,231,428 bp, and 10,229.1 bp to 4,915,960 bp, respectively, which is similar to the quality of the sequences used in other studies focused on S. enterica and Bacillus cereus (13, 25).

**FIG 1:**
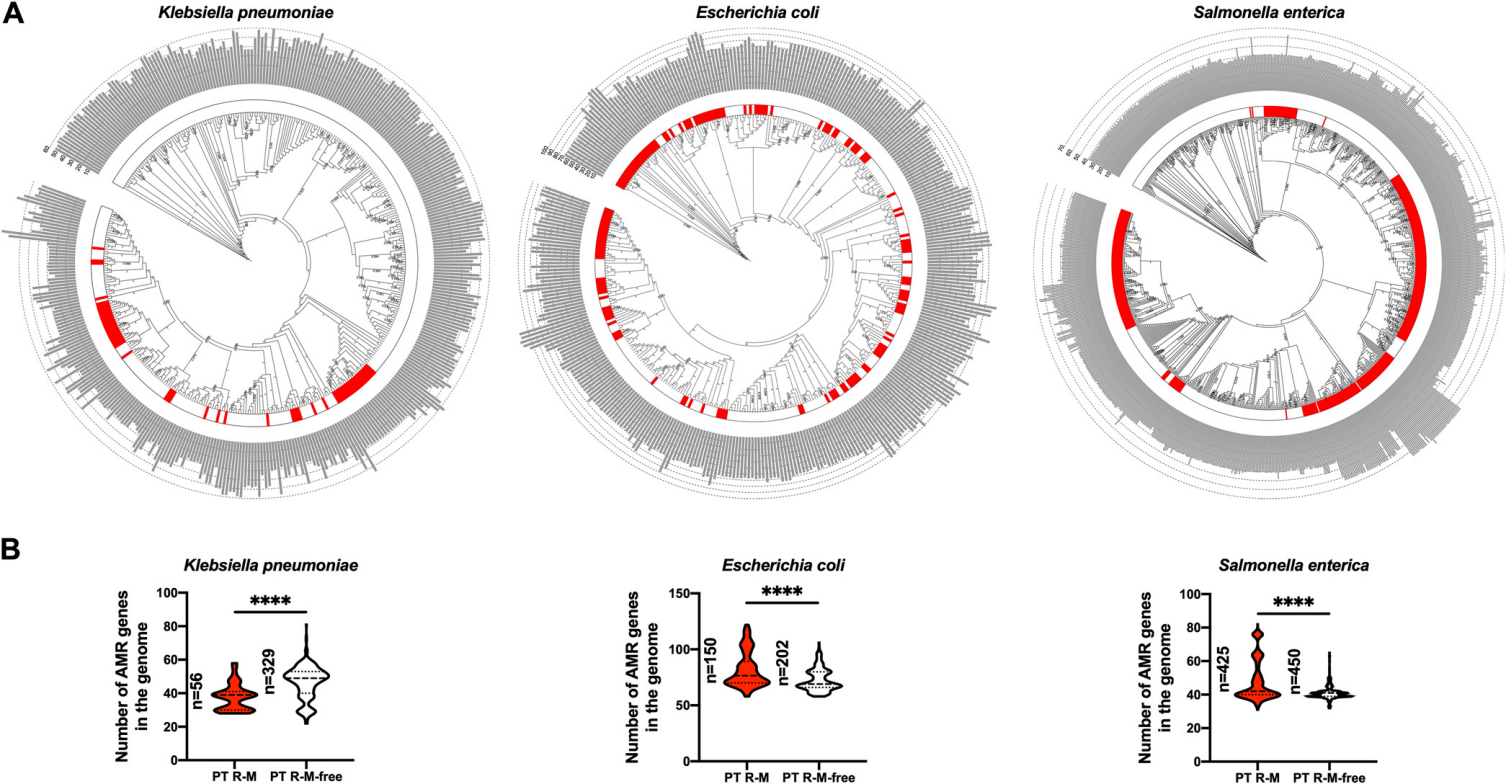
Relationship between the distribution of RGI-predicted AMR genes and PT R-M clusters in bacteria. (A) Core gene-based phylogenetic tree of the three bacteria. The core gene sequences of 385 K. pneumoniae, 352 E. coli, and 875 S. enterica strains were used to construct phylogenetic trees. The value on each branch in the phylogenetic tree is the Shimodaira-Hasegawa (SH) value of the node, which is used to evaluate the reliability of the branch. SH values with values greater than 0.9 are shown in the graph. The inner track next to the tree shows whether the strain on the corresponding leaf harbors the PT R-M system. Red blocks indicate the representation of PT R-M. The outer track shows the total number of AMR genes predicted by RGI in the genome of the strains on the corresponding leaf. (B) Differences in the distribution of AMR genes between strains with and without the PT R-M system in different bacteria. The dashed lines represent the median, and the dotted lines represent the quartile. The significance was analyzed using the two-sample Wilcoxon-Mann-Whitney test (two-tailed; *, *P* < 0.05; **, *P* < 0.01; ***, *P* < 0.001; ****, *P* < 0.0001).

To identify the potential influence of the genetic background on the distribution of PT systems, we constructed phylogenetic trees for the three bacterial groups to analyze the genetic background of the strains with PT R-M in the population based on the core gene alignment results obtained by pangenome analysis (Fig. 1A). The results showed that for the samples analyzed, in K. pneumoniae and E. coli, the strains harboring PT R-M clusters (inner track, red color block) formed discrete phylogenetic clusters, while in S. enterica, the strains harboring PT R-M clusters were more clustered on the phylogenetic tree. However, the strains with PT R-M clusters in these three bacteria were distributed on multiple branches of the phylogenetic tree, indicating that the genetic backgrounds have limited influence on the distribution of PT R-M.

Taking advantage of Resistance Gene Identifier (RGI), we predicted the AMR genes and counted the number of AMR genes in the genome of the strains (Fig. 1A, outermost track; Table S3). Next, we divided the strains into PT R-M and PT R-M-free groups based on the presence and absence of *dnd* gene clusters, respectively, and analyzed the potential differences in the distribution of the total number of AMR genes between the two groups (Fig. 1B). In K. pneumoniae, the median number of AMR genes in the strain harboring PT R-M was 39, while in the PT R-M-defective strain, the median number of AMR genes increased by 25.64% to 49, which indicated that the PT R-M system significantly restricted AMR gene acquisition (Wilcoxon-Mann-Whitney test, *P* < 0.0001). However, the trends in E. coli and S. enterica were different from those we found in K. pneumoniae. For E. coli and S. enterica, the median numbers of AMR genes in the PT R-M group were 76.50 and 42, while the corresponding values were slightly reduced to 69 and 41 in the PT R-M-free group, respectively (Wilcoxon-Mann-Whitney test, *P* < 0.0001).

To further explore whether the effect of PT R-Ms on AMR gene distribution was the same among different classes of AMR genes, we classified AMR genes according to gene family. We found that most of the AMR gene families were ubiquitous in these three bacteria, while 16.36%, 16.36%, and 13.79% of the AMR gene families were unique in K. pneumoniae, E. coli, and S. enterica, respectively (Table S3).

**FIG 2:**
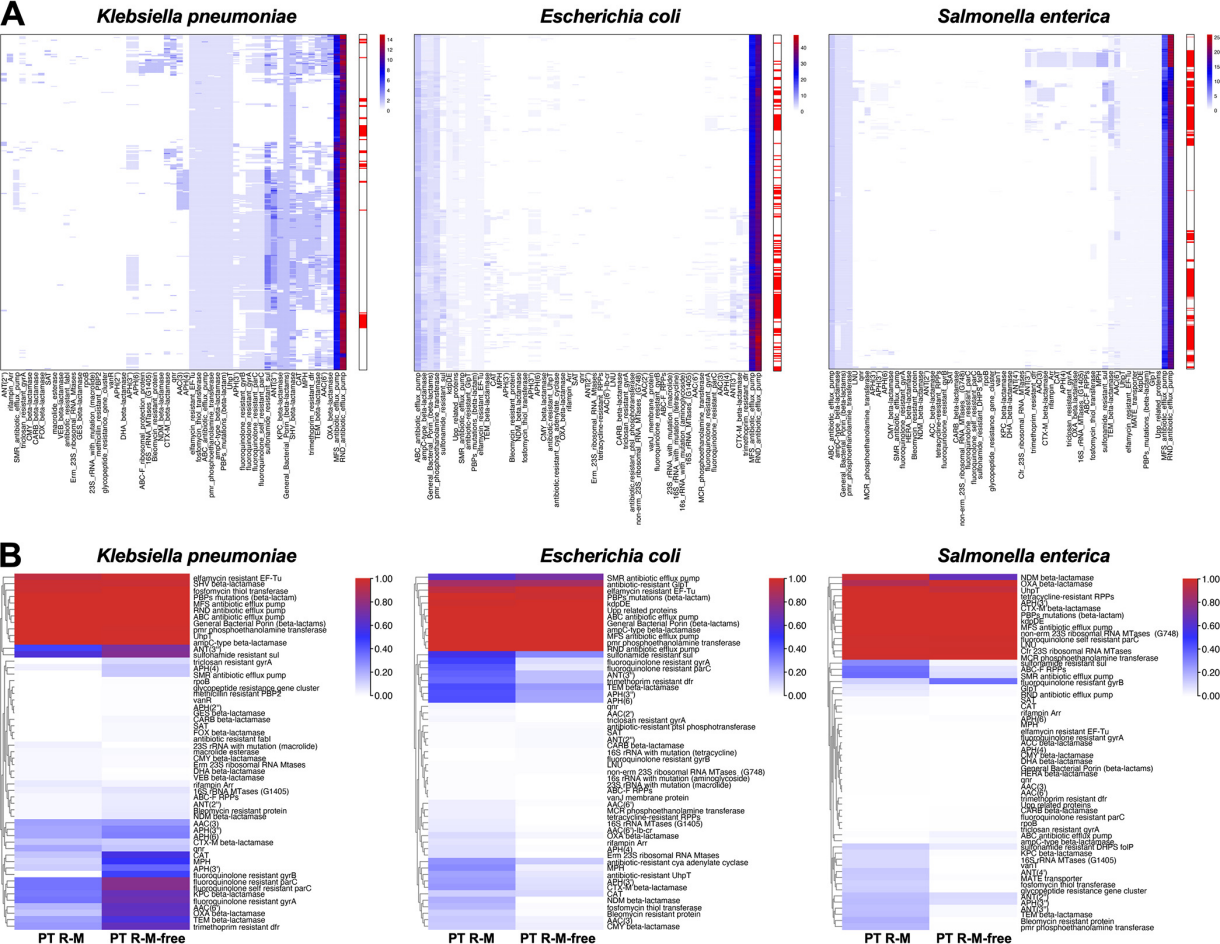
Heatmap of the distribution of the AMR gene families in the genomes. (A) Heatmap of the number distribution of the AMR gene family genes in the genomes. The rows in the heatmap represent strain genomes, and the columns represent gene families. The color of the blocks represents the number of genes in the same gene family; red means the highest value, white means 0, and blue means the middle value. The bar on the right side of the heatmap indicates the existence of the PT R-M system. Strains harboring PT R-M are highlighted with red blocks. (B) Heatmap of the proportion of AMR gene families in the groups between strains with and without the PT R-M system in different bacteria. The rows in the heatmap represent the gene families, and the columns represent two groups of strains that were distinguished by whether they contained the PT R-M system. The red blocks represent 100% occupancy of the gene family in the group, and the white blocks indicate that the gene family does not exist in the group.

Furthermore, heatmaps of the distribution of the AMR gene based on the gene number in each gene family were drawn and clustered according to the similarity of the distribution (Fig. 2A). Except for the MFS antibiotic efflux pump, RND antibiotic efflux pump, and ABC antibiotic efflux pump families, which were present consistently in all the strains, the distribution of the remaining AMR gene families varied among strains. There were obvious differences in the kinds and numbers of AMR gene families among the strains harboring PT R-M clusters, implying that there is no apparent link between PT R-M clusters and the kinds of AMR gene families.

Next, heatmaps were drawn according to the proportions of these families in the PT R-M and PT R-M-free groups (Fig. 2B). The data showed that the proportion of several AMR gene families was close to 100% (shown by the red color block) in the strains, implying that these gene families might not be affected by external factors. For the remaining gene families, the abundance of AMR genes in nearly all branches except six gene families, including 23S rRNA with mutation (macrolide), CTX-M beta-lactamase, DHA beta-lactamase, VEB beta-lactamase, qnr, and rifampin Arr, was negatively correlated with the presence of PT R-M in K. pneumoniae, which suggested that the PT R-M system restricts AMR gene acquisition in a nonselective manner.

In sharp contrast to the phenomenon we found that in K. pneumoniae, only nine families, including 16S rRNA with mutation (tetracycline), ANT(2″), CARB beta-lactamase, antibiotic-resistant GlpT, antibiotic-resistant PtsI phosphotransferase, elfamycin-resistant EF-Tu, fluoroquinolone-resistant GyrB, SMR antibiotic efflux pump, and SAT, showed a negative correlation with the existence of the PT R-M system in E. coli. This situation became more pronounced for S. enterica, in which only three families, ABC antibiotic efflux pump, AmpC-type beta-lactamase, and fluoroquinolone-resistant GyrB, followed the rule exhibited in K. pneumoniae, while the rest of the families were all positively correlated with the existence of the PT R-M system.

Together, these data showed that the presence of PT R-Ms was negatively associated with the distribution of AMR genes in the genomes of K. pneumoniae, but the effect was the opposite in E. coli and S. enterica.

### Me R-M, CRISPR-Cas, and the genetic context do not contribute to the various impacts of PT R-M systems on the distribution of AMR genes.

**FIG 3:**
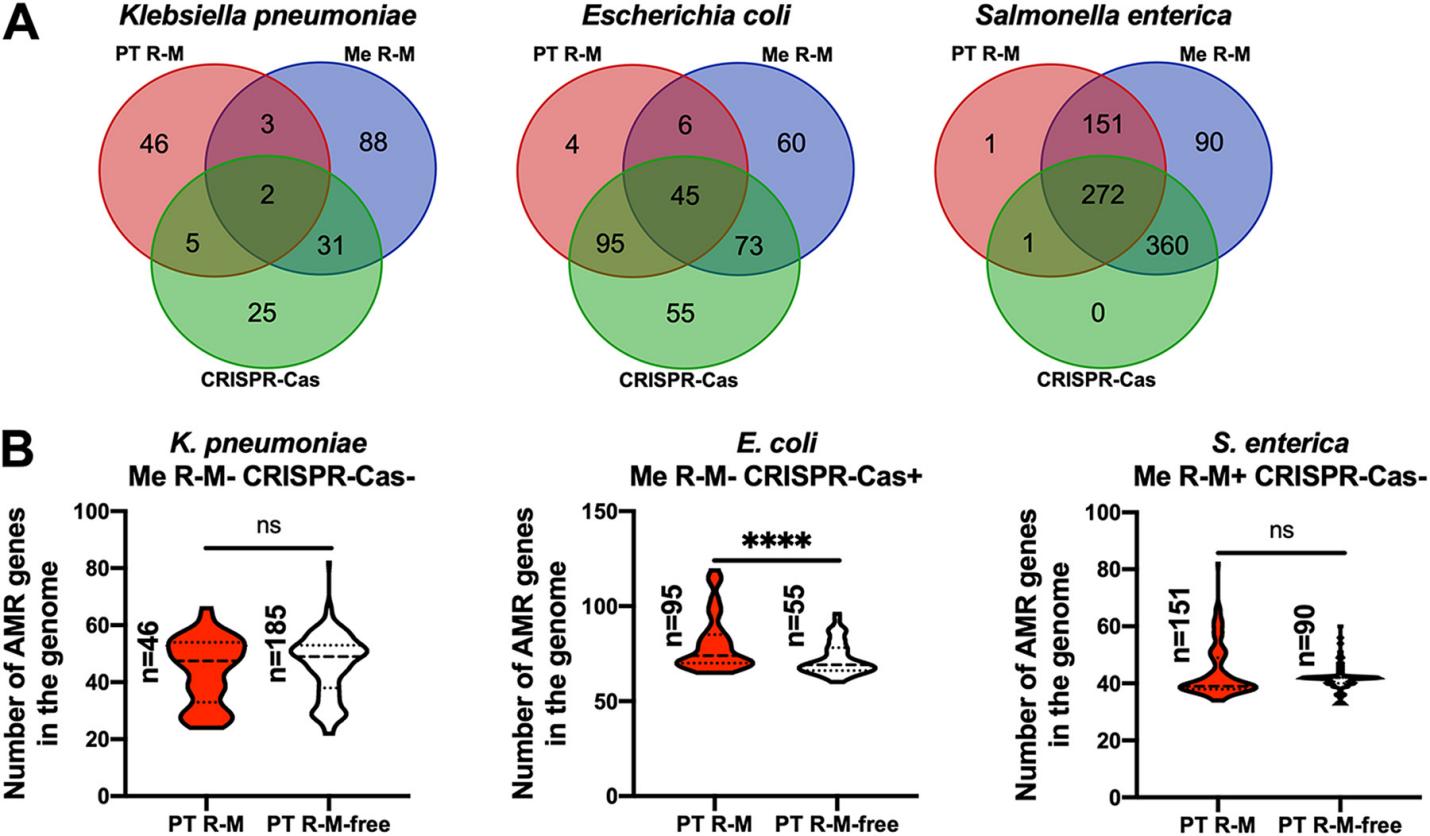
Relationship between the presence of Me R-M and CRISPR-Cas and the effects of PT R-M systems on the distribution of AMR gene numbers. (A) Venn diagram of the distribution of PT R-M, Me R-M, and CRISPR-Cas in the strains of K. pneumoniae, E. coli, and S. enterica. (B) Differences in the number distribution of AMR genes between the PT R-M group and the PT R-M-free group in strains with different Me R-M and CRISPR-Cas statuses. The dashed lines represent the median, and the dotted lines represent the quartile. The significance was analyzed using the two-sample Wilcoxon-Mann-Whitney test (two-tailed; *, *P* < 0.05; **, *P* < 0.01; ***, *P* < 0.001; ****, *P* < 0.0001).

Due to the widespread of other defense systems, such as Me R-M and CRISPR-Cas, in bacteria, which have been proven to influence the distribution of AMR genes, the inconsistencies we found in these three bacteria might be caused by the different distributions of Me R-M and CRISPR-Cas systems. To rule out this possibility, we analyzed the presence of Me R-M and CRISPR-Cas systems using Restriction-ModificationFinder (25) and CRISPRCasFinder (26) (Fig. 3A; Table S4). In contrast to K. pneumoniae, where 60% of the strains do not have either Me R-M or CRISPR-Cas systems, most strains in E. coli (334 out of 352) and S. enterica (874 out of 875) had at least one of these two defense barriers. To rule out the potential influence of Me R-M and CRISPR-Cas, the simplest approach is to exclude bacteria containing these two systems. Thus, we analyzed the distribution of AMR genes in the PT R-M group and PT R-M-free group in K. pneumoniae lacking both Me R-M and CRISPR-Cas. Although the median and lower quartile of the data in the PT R-M group were smaller than those in the PT R-M-free group, there was no statistically significant difference between the two groups (*P* = 0.3935) (Fig. 3B). To this end, we calculated the covariance value, which represents the relationship between two random variables, to measure the directional relationship between the existence of the PT R-M system and the distribution of AMR genes. The covariance analysis for the data above showed that the covariance value between the PT R-M group and the PT R-M-free group was −0.284383726, implying a negative correlation between the presence of the PT R-M system and the number of AMR genes, which is consistent with the conclusion we reached in the previous section of results.

However, it is not suitable to exclude both CRISPR-Cas and Me R-M in E. coli or S. enterica, respectively, due to the limitation of the sample size. Thus, we analyzed the influence of PT R-M on the AMR distribution in E. coli lacking Me R-M while maintaining CRISPR-Cas and in S. enterica lacking CRISPR-Cas while harboring Me R-M. For E. coli, the presence of PT R-M increased the number of AMR genes (*P* < 0.0001), and for S. enterica, a larger extreme value was found in the PT R-M group (*P* = 0.1519), and the covariance value between the two groups was 0.603766819, which implied a positive correlation between the presence of the PT R-M system and the number of AMR genes (Fig. 3B). Thus, the inconsistencies we found in the three bacteria are unlikely to be caused by the distribution of Me R-M or CRISPR-Cas.

**FIG 4:**
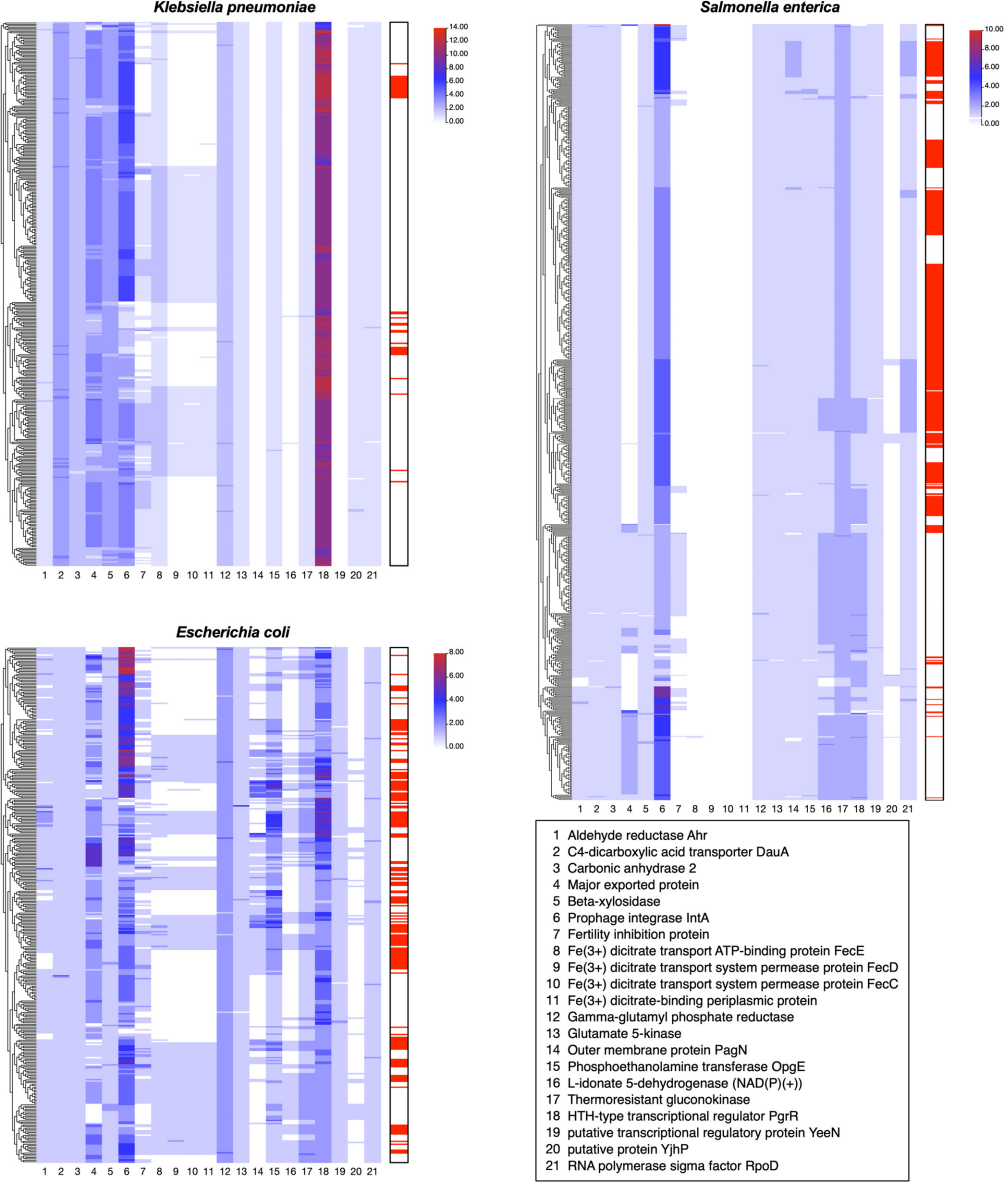
Heatmap of the distribution of 21 major genes near the *dnd* cluster in the genomes of K. pneumoniae, E. coli, and S. enterica. The rows in the heatmap represent strain genomes; the columns represent the genes near the *dnd* cluster. The color of the blocks represents the number of genes in the same function; red indicates the highest value, white indicates 0, and blue indicates the middle value. The bar on the right side of the heatmap indicates whether the corresponding strain harbors the PT R-M system. Strains harboring PT R-M are highlighted with red blocks.

In addition, we explored whether there were some colocalized genes upstream and downstream of the PT R-M clusters that affected the distribution of AMR genes. To this end, we set a 5,000-bp region upstream and downstream of PT R-M clusters for scanning. We screened 54, 117, and 394 upstream sequences along with 54, 129, and 379 downstream sequences fulfilling this sequence length from the genomic data of K. pneumoniae, E. coli, and S. enterica, respectively. Prokka was used for gene annotation, and the presence/absence matrix of the upstream and downstream gene distributions of the PT R-M gene cluster in the genome was plotted for each strain and clustered according to the similarity of the gene distribution (Fig. S1; Table S5). Genes with higher frequencies upstream and downstream of the PT R-M cluster were selected for further analysis. In addition, 21 types of genes could be identified according to the function of the encoded protein. For these genes, heatmaps of the number of proteins distributed in the genome were drawn and clustered according to the similarity of protein distribution based on the three bacterial pangenome analysis data sets (Fig. 4; Table S5). The data showed that most proteins were universal in the three bacteria, which was consistent with a previous study focused on E. coli, suggesting that most open reading frames (ORFs) outside the *dnd* cluster were present in the bacterial genomes, ignoring the existence of the *dnd* cluster (27). Only eight proteins, including Fe^3+^ dicitrate transport ATP-binding protein FecE, Fe^3+^ dicitrate transport system permease protein FecD, Fe^3+^ dicitrate transport system permease protein FecC, Fe^3+^ dicitrate-binding periplasmic protein, outer membrane protein PagN, l-idonate 5-dehydrogenase [NAD(P)(+)], putative transcriptional regulatory protein YeeN, and putative protein YjhP, were differentially distributed in K. pneumoniae, E. coli, and S. enterica (Table S5).

Among them, FecCDE and Fe^3+^ dicitrate-binding periplasmic protein are related to bacterial iron transport (28, 29), PagN is related to bacterial invasion of host cells (30, 31), and l-idonate 5-dehydrogenase [NAD(P)(+)] is involved in l-iodic acid catabolism pathways (32), while *yeeN* (which encodes a conserved hypothetical protein) and *yjhP* (which encodes a putative methyltransferase) are most commonly found at the 3′ end of tRNA*^leuX^* islands (33). In addition, YjhP may be associated with bacterial resistance (34, 35). However, there is no evidence for these proteins to be related to the acquisition of AMR genes.

In conclusion, the distribution of Me R-M and CRISPR-Cas in bacteria, as well as the genes upstream and downstream of the PT R-M cluster, do not contribute to the impacts of PT R-M systems on the distribution of AMR genes in K. pneumoniae, E. coli, and S. enterica.

### The PT R-M system is associated with fewer plasmid- and prophage-derived AMR genes.

Since bacteria can acquire AMR genes in a variety of ways, we speculated that these inconsistencies might be due to the different origins of AMR genes. For example, mutated drug binding sites that seemed unlikely to be affected by the status of PT R-M systems were also predicted by the RGI tool, which may interfere with the analysis. According to research, AMR genes derived from HGT are more susceptible to bacterial defense systems (10, 11, 13). Because the PT R-M system is a novel bacterial defense barrier against invading genetic parasites, such as phages and plasmids (14–16), we proposed that the PT R-M system might influence the host to acquire AMR genes by interfering with the HGT process, as mobile genetic elements (MGEs), plasmids, and prophages are important pathways for HGT of bacterial AMR genes (2, 8, 9). Therefore, the presence of the PT R-M system might have an impact on the distribution of plasmid- and prophage-derived AMR genes (plasmid-AMR genes, prophage-AMR genes) in bacteria. To verify this hypothesis, we first analyzed the relationship between AMR genes and plasmids in bacteria.

**FIG 5:**
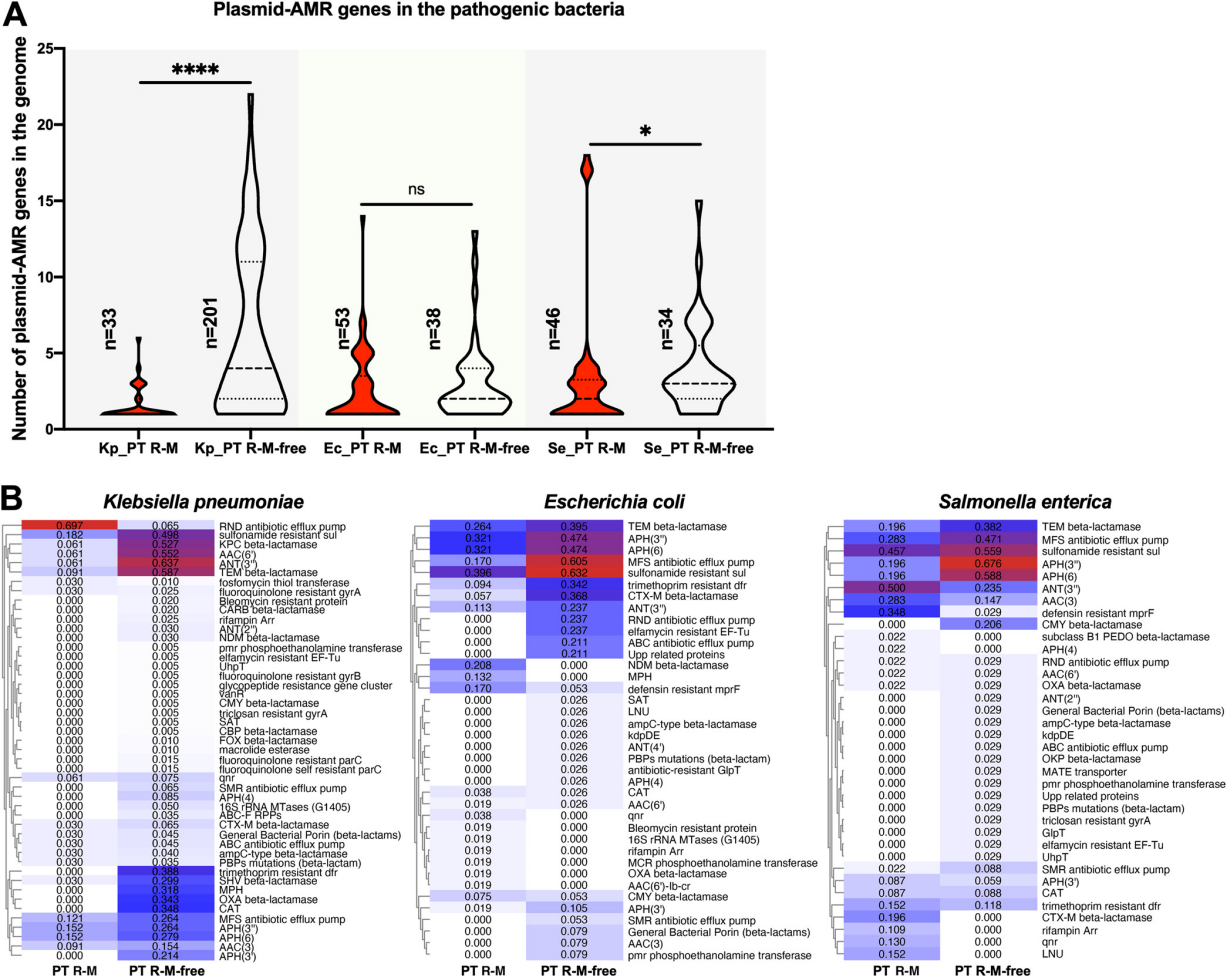
Distribution of plasmid-AMR genes among selected pathogenic bacteria. (A) Violin plot showing the distribution of the plasmid-AMR gene numbers in the bacterial genome. The dashed lines represent the median, and the dotted lines represent the quartile. Kp, Ec, and Se represent K. pneumoniae, E. coli, and S. enterica, respectively. The significance was analyzed using two-sample Wilcoxon-Mann-Whitney tests between the groups with or without PT R-M in the same bacterium (two-tailed; *, *P* < 0.05; **, *P* < 0.01; ***, *P* < 0.001; ****, *P* < 0.0001; ns, not significant). (B) Heatmap of the proportion of AMR gene families related to plasmids in the groups between strains with and without the PT R-M cluster in different bacteria. The red blocks represent 100% occupancy of the gene family in the group, and the white blocks indicate that the gene family does not exist in the group. The values in the color blocks represent the fractional proportion of the corresponding gene family in the population.

PlasmidFinder v2.1 was used to predict plasmid replicons in each individual contig. Since K. pneumoniae, E. coli, and S. enterica all belong to *Enterobacteriaceae*, it is appropriate to use the *Enterobacteriales* database as the query. There were 33, 53, and 46 strains of K. pneumoniae, E. coli, and S. enterica, respectively, that were found to harbor plasmids in the PT R-M group. In the PT R-M-free group, the corresponding values were 201, 38, and 34, respectively (Table S6). Then, the distribution of the number of plasmid-AMR genes in these strains was calculated (Fig. 5A; Table S6). For K. pneumoniae and S. enterica, the number distribution of plasmid-AMR genes in the PT R-M group was significantly lower than the corresponding values in the PT R-M-free group (*P* < 0.0001 and *P* = 0.0273, respectively). For E. coli, a lower median and upper quartile were identified in the PT R-M group (*P* = 0.1836), and the covariance value between the PT R-M group and the PT R-M-free group was −0.115565753, indicating a negative correlation between the presence of the PT R-M system and the number of plasmid-AMR genes in the genomes. For K. pneumoniae and S. enterica, the covariance values between the PT R-M group and the PT R-M-free group were −0.587113741 and −0.138125, respectively.

Furthermore, we analyzed the differences in the distribution of plasmid-AMR genes according to AMR gene families between the PT R-M group and the PT R-M-free group in these three bacteria. As previously described, the proportions of different gene families in each group were calculated separately (Fig. 5B). The heatmap showed that for K. pneumoniae, E. coli, and S. enterica, there were fewer kinds of gene families when PT R-M existed (19 versus 47, 22 versus 29, and 21 versus 30, respectively). In addition, 44 of 47, 25 of 38, and 25 of 36 gene families in K. pneumoniae, E. coli, and S. enterica, respectively, were negatively correlated with the presence of the PT R-M system. These results demonstrated that PT R-M could inhibit the distribution of plasmid-AMR genes.

Next, the distribution of prophage-AMR genes in bacterial genomes was identified. Although prophage sequences have been identified in more than half of the genomes of K. pneumoniae, E. coli, and S. enterica with the help of PhiSpy (36) (Table S7), few bacterial genomes carrying prophage-AMR genes were detected. For K. pneumoniae, E. coli, and S. enterica, there were 0, 20, and 1 strain harboring prophage-AMR genes in the PT R-M group, respectively, while in the PT R-M-free group, the corresponding values were 14, 44, and 12. Consistent with the small number of prophages carrying AMR genes, the prophage-AMR genes in the eligible genomes were few in number, ranging from 1 to 5 (Table S7). The proportion of strains with prophage-AMR genes in PT R-M was lower than that in the PT R-M-free group in K. pneumoniae (0 versus 4.26%), E. coli (13.33% and 21.78%), and S. enterica (0.24% and 2.67%), suggesting a negative correlation between the presence of the PT R-M system and the acquisition of prophage-AMR genes in the genomes. Together, these data implied that the presence of PT R-M could reduce the number of AMR genes derived from plasmids and prophages.

### The presence of the PT R-M system is responsible for a lower number of integrating MGE-related AMR genes.

In addition to plasmids and prophages, integrating MGEs (iMGEs), including insertion sequences (ISs), unit transposons (Tns), and integrative conjugative elements (ICEs), are also involved in the HGT process of AMR genes (7, 37). Therefore, we explored the potential impact of PT R-M on the distribution of AMR genes associated with integrating MGEs (iMGE-AMR genes). MobileElementFinder v1.0.3 was used to perform the prediction of iMGEs in the bacterial genomes. Then, RGI prediction was performed on these iMGEs, identifying the iMGE-AMR genes, and the proportions of the strains with iMGE-AMR genes in the bacteria were calculated. Not surprisingly, iMGEs were ubiquitous in K. pneumoniae, E. coli, and S. enterica. Nearly all the strains contained at least one iMGE on the genome, except for one K. pneumoniae strain (Kleb_pneu_UCI_60_V1; Assembly accession no. GCF_000688195) and one S. enterica strain (W41; GCF_000376525).

In contrast to the wide distribution of iMGEs, only 21.43% of K. pneumoniae strains, 28.67% of E. coli strains, and 14.82% of S. enterica strains were found to harbor iMGE-AMR genes in their genomes in the PT R-M group. The values in the PT R-M-free group were 75.08%, 35.64%, and 4.89%, respectively.

**FIG 6:**
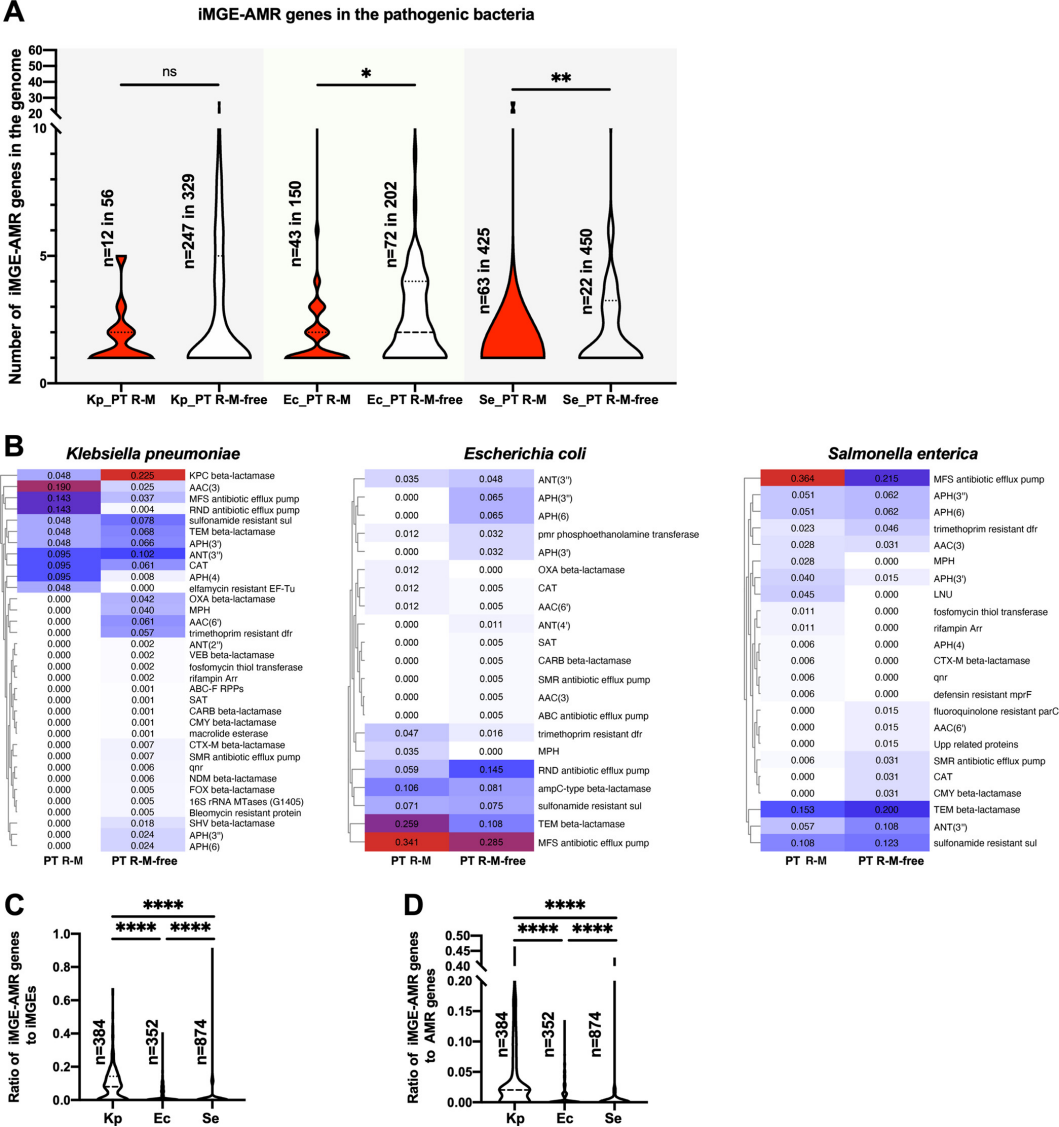
Distribution of iMGE-AMR genes among selected pathogenic bacteria. (A) Violin plot showing the distribution of iMGE-AMR gene numbers in the bacterial genome. The dashed lines represent the median, and the dotted lines represent the quartile. Kp, Ec, and Se represent K. pneumoniae, E. coli, and S. enterica, respectively. (B) Heatmap of the proportion of AMR gene families related to iMGE in the groups between strains with and without the PT R-M system in different bacteria. The red blocks represent 100% occupancy of the gene family in the group, and the white blocks indicate that the gene family does not exist in the group. The values in the color blocks represent the fractional proportion of the corresponding gene family in the population. (C) Violin plot showing the distribution of the iMGE-AMR gene to iMGE ratios in the bacterial genome. The significance was analyzed using two-sample Wilcoxon-Mann-Whitney tests between the groups with or without PT R-M in the same bacterium. (D) Violin plot showing the distribution of the iMGE-AMR gene to total AMR ratios in the bacterial genome. The significance was analyzed using two-sample Wilcoxon-Mann-Whitney tests between the groups with or without PT R-M in the same bacterium (two-tailed; *, *P* < 0.05; **, *P* < 0.01; ***, *P* < 0.001; ****, *P* < 0.0001).

Based on the above-described data, the distribution of the iMGE-AMR gene was determined (Fig. 6A; Table S8). The results showed that the number of iMGE-AMR genes in the E. coli strains and S. enterica strains harboring the PT R-M system was significantly less than that in the PT R-M-free strains (*P* = 0.0312 and *P* = 0.0050, respectively), while for K. pneumoniae strains, the upper quartiles and the extreme value in the PT R-M group were both smaller than those in the PT R-M-free group (*P* = 0.2245). The covariance analysis for the data above showed that the covariance values between the PT R-M group and the PT R-M-free group in K. pneumoniae, E. coli, and S. enterica were −0.090293824, −0.142003781, and −0.030865052, respectively, which implied a negative correlation between the presence of the PT R-M system and the number of iMGE-AMR genes in the genomes.

Furthermore, we analyzed the differences in the distribution of iMGE-AMR genes in AMR gene families between the PT R-M group and the PT R-M-free group in these three bacteria (Fig. 6B). As previously described, the proportions of different gene families in each group were calculated separately. The heatmap showed that for K. pneumoniae and E. coli, the number of gene families to which iMGE-AMR genes belonged in the PT R-M groups was less than that in the PT R-M-free groups (11 versus 33 and 12 versus 19, respectively). In addition, 23 out of 34 and 12 out of 21 gene families in K. pneumoniae and E. coli, respectively, were negatively correlated with the presence of the PT R-M system. For S. enterica, although the number of gene families to which iMGE-AMR genes belonged in the PT R-M group was more than the corresponding value in the PT R-M-free group (18 versus 15), more than half of the gene families (12 in 23) were negatively correlated with the presence of the PT R-M system.

Moreover, based on the above-described data, we attempted to analyze the preference for iMGE-related AMR genes in these three bacteria. Data for the PT R-M groups and the corresponding PT R-M-free groups were collected together to observe overall trends. We calculated the proportion of iMGE-AMR genes in iMGEs and total AMR genes in each strain separately and calculated the distribution of the above-described ratios in the three bacteria (Fig. 6C and D; Table S8). The results showed that both ratios in K. pneumoniae were much higher than the corresponding values in E. coli or S. enterica (*P* < 0.0001). In the experiment, although the two ratios in S. enterica showed a significant difference from the corresponding results in E. coli (*P* < 0.0001), in fact, both medians of the corresponding data in the two bacteria were 0, and the data in E. coli had larger upper quartiles, while the data in S. enterica had larger extreme values. This result implied that K. pneumoniae may be more inclined to acquire AMR genes through iMGE-mediated HGT than the other two bacteria, which makes K. pneumoniae more sensitive to the PT R-M system and, to some extent, explains the inconsistencies we encountered earlier. In summary, these data suggested that the presence of PT R-M could reduce the number of AMR genes derived from integrating MGEs, and the effect may be influenced by the preference for iMGE-AMR genes in bacteria.

### The PT R-M system is associated with a lower frequency of HGT.

**FIG 7:**
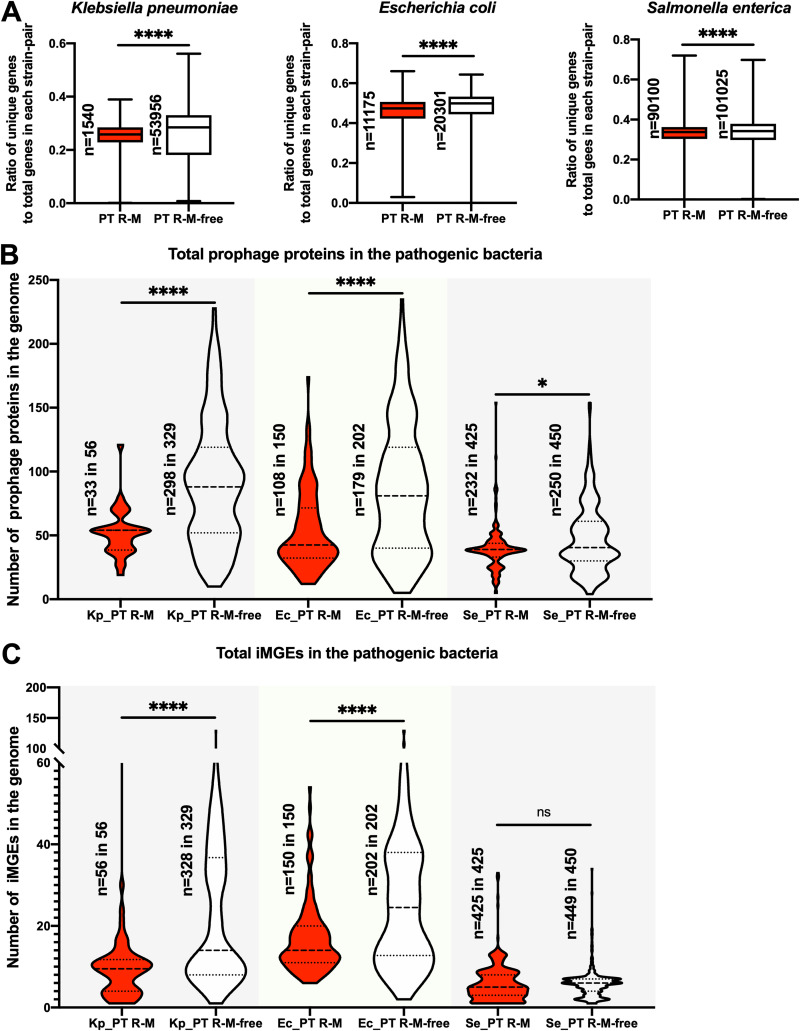
Distribution of unique genes and mobile genetic elements among selected pathogenic bacteria. (A) Comparison of unique gene diversity values for total genes among the strains with PT R-M or without PT R-M. (B) Violin plot showing the distribution of prophage protein numbers in the bacterial genome. The dashed lines represent the median, and the dotted lines represent the quartile. Kp, Ec, and Se represent K. pneumoniae, E. coli, and S. enterica, respectively. The significance was analyzed using a two-sample Wilcoxon-Mann-Whitney test between the groups with or without PT R-Ms in the same bacterium (two-tailed; *, *P* < 0.05; **, *P* < 0.01; ***, *P* < 0.001; ****, *P* < 0.0001). (C) Violin plot showing the distribution of iMGE numbers in the bacterial genome. The dashed lines represent the median, and the dotted lines represent the quartile. The significance was analyzed using a two-sample Wilcoxon-Mann-Whitney test between the groups with or without PT R-Ms in the same bacterium (two-tailed; *, *P* < 0.05; **, *P* < 0.01; ***, *P* < 0.001; ****, *P* < 0.0001).

Since AMR genes carried by MGE spread across strains via HGT (6, 7), we attempted to determine whether the suppression of MGE-derived AMR gene distribution by PT R-M was associated with a reduction in HGT frequency. First, we analyzed the variation in unique genes of strains with or without the PT R-M system in the bacteria according to the results of pangenome analysis (Fig. 7A; Table S9). The results showed that in all three kinds of species, the ratio of unique genes was significantly reduced by the PT R-M system (*P* < 0.0001). Furthermore, differences in the number distribution of prophage proteins in the genome between the PT R-M group and the corresponding PT R-M-free group in bacteria were assessed (Fig. 7B; Table S7). The data showed that in K. pneumoniae, E. coli, and S. enterica, the number from the PT R-M group was significantly lower than that from the PT R-M-free group (*P* < 0.0001, *P* < 0.0001, and *P* = 0.0189, respectively), suggesting that the presence of PT R-M restricted the number of prophage genes in the genomes. This result is consistent with the fact that PT R-M could protect the host from the invasion of phages (15). In addition, the distribution of the number of iMGEs in the strains was determined (Fig. 7C; Table S8). The results demonstrated that the number of iMGEs in the K. pneumoniae and E. coli strains with PT R-M was significantly less than that in the corresponding strains without PT R-M (*P* < 0.0001, respectively), which is consistent with the covariance analysis (−1.585828993 and −2.792210098, respectively). However, the number of iMGEa in the S. enterica strains with PT R-M was not significantly different from that in the corresponding strains without PT R-M (*P* = 0.1482). Together, our results suggest that the PT R-M system reduces MGE-mediated HGT frequency within K. pneumoniae and E. coli, but the effect was limited in S. enterica.

### PT R-M clusters and the core genes of bacteria showed no coevolutionary relationship.

The acquisition of AMR genes is an evolutionary behavior in bacteria. We suspect that the PT R-M may not be closely related to the evolution of bacteria since there was only a small proportion of strains carrying the PT system among the above-described pathogens. This out-of-sync phenomenon might also contribute to the inconsistencies we found previously. Based on the results of the pangenome analysis, we attempted to show the evolutionary relationship between the PT R-M gene cluster and bacterial core genes.

**FIG 8:**
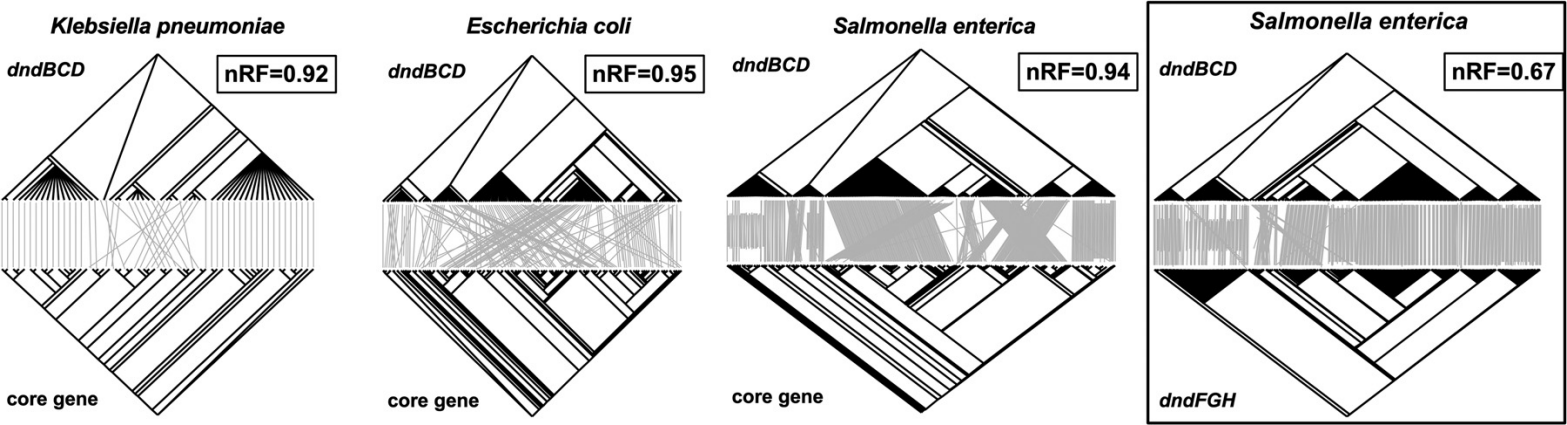
Coevolutionary analysis between bacterial *dndBCD* clusters and corresponding core genes. The upper and lower halves of the tanglegram plot are the phylogenetic tree of the *dndBCD* clusters and the core genes of the corresponding strains, respectively. Shown in the top right box of the tanglegram plot is the normalized Robinson-Foulds distance between the two phylogenetic trees. The results of coevolutionary analysis of the Salmonella *dndBCD* and *dndFGH* phylogenetic trees were used as a reference (marked by black boxes).

The phylogenetic trees for the core genes of bacteria with PT R-M were constructed, and the topological structure was compared with the corresponding phylogenetic trees of the *dndBCD* cluster by the normalized Robinson-Forth (nRF) distance (Fig. 8). The nRF value is between 0 and 1, and a lower value means a more similar topology between the two phylogenetic trees being compared.

In a previous study, we indicated that there was coevolution between *dndBCD* and *dndFGH* in S. enterica (20). Thus, we used it as a reference here. The results showed that the nRF value between *dndBCD* and *dndFGH* in S. enterica was less than 0.7. However, as shown in Fig. 8, the nRF value between *dndBCD* and the core gene was 0.92, 0.95, and 0.94 in K. pneumoniae, E. coli, and S. enterica, respectively, all of which were much larger than 0.7 and were very close to 1.

Based on the reference values, there might not be a coevolutionary relationship between PT R-M clusters and the core genes of bacteria. This result was consistent with reports that the PT R-M system is transmitted in bacteria via HGT (38). Therefore, the temporal order in which PT R-M and HGT-related AMR genes appear in the strain genome may affect the restriction effect provided by PT R-M on the horizontal transfer of AMR genes. This might be another factor that interferes with the effect of PT R-M on suppressing the distribution of AMR genes.

## DISCUSSION

Currently, bacterial AMR is a common problem faced by clinicians worldwide. While the causes of bacterial AMR are complex, it is worth mentioning that HGT of AMR genes is one of the main pathways for bacteria to acquire AMR elements (2). Several studies have shown that the defense system represented by Me R-M systems or CRISPR-Cas systems can interfere with AMR gene acquisition through HGT (10, 13).

PT modification has recently been shown to have the potential to regulate gene expression in bacteria and could form a defense system to prevent invasion by foreign DNA, such as plasmids and phages or viruses (14–16, 39–41), which prompted us to explore the potential impact of PT R-M systems on bacterial antimicrobial resistance.

In the process of searching for antimicrobial-resistant bacteria harboring PT gene clusters, we found that the existence of PT-based defense systems may influence the distribution of AMR genes in the genomes of the strains. For example, among the 3,970 strains of A. baumannii, we found that there were only three strains, RUH1486, XH694, and NIPH146, harboring PT R-M gene clusters (see Table S1 in the supplemental material). Multilocus sequence typing (MLST; Pasteur) analysis showed that RUH1486 and NIPH146 belong to ST25, and XH694 belongs to ST106. RGI analysis showed that compared to the typical multiple-drug resistance (MDR) strains MDR-ZJ06, ACICU, AB0057, and AYE (42–45), the families and number of AMR genes in the above-mentioned strains with PT R-M clusters were fewer and were similar to those in the typical antibiotic-sensitive strains SDF and LUH7841 (46, 47). In addition, compared to RUH1486 and NIPH146, NM3, LUH6220, and 4390, which also belong to ST25, had more AMR genes (Table S1) (47).

These phenomena implied that the existence of the PT R-M system might reduce the abundance of AMR genes in the genome and prompted us to turn to K. pneumoniae, E. coli, and S. enterica, which have a much larger sample size. However, we failed to observe a consistent negative correlation between the existence of the PT R-M system and the distribution of AMR genes. After ruling out the potential effects caused by the coexisting Me R-M and CRISPR-Cas in the genome, as well as the colocated genes from the 5,000-bp genetic context upstream and downstream of PT R-M clusters, we suspected that these inconsistencies might be caused by the origin of AMR genes. Following this idea, we focused our attention on HGT-derived AMR genes. As expected, PT R-M systems repressed the acquisition of HGT-derived AMR genes in all three bacteria, similar to the Me R-M system or CRISPR-Cas system. In addition, the cause of this suppression phenomenon was determined to be due to a drop in HGT frequency.

Studies have shown that the Me R-M system and the CRISPR-Cas system limit the antibiotic resistance of K. pneumoniae and E. coli (12, 48–50). However, for S. enterica, although CRISPR-Cas has been shown to be associated with antimicrobial resistance (51), the Me R-M system has limited impact on genetic evolution (25). The differences in the effect of bacterial defense systems on bacterial resistance between bacteria may be related to the characteristics of the bacteria themselves. For A. baumannii and E. coli, researchers found that they have the ability to naturally transform, which is more conducive to the horizontal transfer of AMR genes (52, 53). However, Salmonella does not have the ability to naturally transform, and the transfer of genetic material occurs mainly through conjugation (54). Moreover, a study showed that the barriers imposed by Me R-M systems in E. coli in the transfer of conjugational plasmids were not absolute (55). In our experiments, we also found that there was no significant difference in the distribution of iMGE numbers between the PT R-M group and the PT R-M-free group of S. enterica (Fig. 7C), suggesting that as an R-M system, PT R-M, although able to limit the distribution of iMGE-AMR genes, which are involved in bacterial tolerance to environmental stress and are directly related to bacterial survival, had a limited impact on the distribution of whole iMGE in S. enterica.

For K. pneumoniae, although it does not have the ability to naturally transform (56), it seems that K. pneumoniae is particularly willing to acquire AMR genes by means of HTG. Most of the antimicrobial resistance observed in K. pneumoniae is associated with the accessory AMR genes acquired by HTG rather than mutations in chromosomal genes (57–59). K. pneumoniae may have a stronger ability to take up and/or maintain plasmids. Many different plasmids have been sequenced from K. pneumoniae, which vary widely in length and type of incompatibility. Currently, researchers have isolated K. pneumoniae carrying 4 to 6, or even 10, different plasmids, which allows K. pneumoniae to accommodate more plasmids than E. coli and other Gram-negative ESKAPE pathogens (60, 61). Whole-genome sequencing research on CRKp led to the discovery of K. pneumoniae as the donor and recipient of AMR gene transfer, playing a leading role in the transfer of plasmids and MGEs in hospital outbreaks (61–65). In this study, we also found that the proportion of the iMGE-AMR gene to MGE or AMR in K. pneumoniae was higher than the corresponding values in the other two bacteria (Fig. 6C and D).

Since the effect of Me R-M on the conjugation process may be limited, in the future, it may be considered to explore the relationship between the PT R-M and the distribution of AMR genes in more bacteria that have natural transformation ability, such as Streptococcus pneumoniae, Haemophilus influenzae, Vibrio vulnificus, and the *Neisseria* genus, including N. meningitidis (66–69).

Moreover, a series of coevolutionary analyses between *dnd* gene clusters and bacterial core genes revealed that there was no obvious coevolution between the PT R-M clusters and core genes in the bacteria (Fig. 8). This phenomenon was consistent with reports that *dnd* gene clusters were located on mobile elements such as gene islands and thus spread among strains via HGT (38). We speculated that this may also be one of the factors limiting the effect of PT R-M on the HGT of AMR genes since the PT R-M clusters obtained by HGT cannot influence the AMR genes already present in the strain. In addition, although the PT systems with intact *dnd* clusters collected thus far are all active in the strains, the presence of a complete *dndBCDE*-*FGH* gene cluster does not necessarily mean that the system is functional, which might interfere with the result (40, 70). On the other hand, for bacteria such as S. enterica with a limited HGT pathway (25), the presence of PT R-M in the strains may indicate that they have a relatively stronger ability to acquire foreign genes compared with their counterparts and are more prone to HGT and acquire more MGEs. These factors may all interfere with the role of PT R-M in the genetic evolution of antimicrobial resistance in bacteria.

Interestingly, we found that some hypothetical proteins with amino acid sequences ranging from 34 amino acids (aa) to 359 aa in length, sharing no similarity of sequences or structures from known proteins, are close to the PT R-M system (Table S5). An online BLAST analysis using the NCBI database revealed that all these sequences were also present in strains of the same bacterium without the PT R-M gene cluster, as well as in several other species of bacteria. One sequence-tagged Kp_DndF_UP_group_15 has a particular tendency to be present in K. pneumoniae harboring PT R-M. It might be interesting to explore the potential relationship between Kp_DndF_UP_group_15 and PT R-M systems in K. pneumoniae.

In conclusion, the evolution of bacterial antimicrobial resistance is a complex process influenced by the environment, its own genetic characteristics, and bacterial defense systems. In this process, the PT R-M system based on the *dnd* gene cluster played a certain inhibitory role in the horizontal transfer of AMR genes. This study reveals the role of the PT R-M system in bacterial AMR for the first time and provides a deeper understanding of the diversities in the relationship between the R-M system and the distribution of AMR genes in bacteria.

## MATERIALS AND METHODS

### Genomes.

The genome assembly RefSeq files of the pathogenic strains for bioinformatics analysis, including 8,873 strains of Klebsiella pneumoniae, 22,747 strains of Escherichia coli, 11,354 strains of Salmonella enterica, and 3,970 strains of Acinetobacter baumannii, were all collected from the NCBI database (https://www.ncbi.nlm.nih.gov/) (see Table S1 in the supplemental material).

### Retrieval of strains with PT gene clusters.

Local BLAST v2.9.0 was used for BLAST analysis, with an E value of ≤10^−10^. BLAST hits with aligned lengths of ≤30% of the query proteins were discarded. S. enterica serovar Cerro 87 DndBCDE (GenPept accession nos. ADN26581 to ADN26584) and DndFGH (GenPept accession nos. ADN26586 to ADN26588) were used as queries for the PT modification gene cluster and restriction gene cluster. The genome assembly RefSeq files described above were collected from the NCBI database as the subjects, and the TBLASTN command was executed.

According to the BLAST comparison results, the strains with complete *dndBCDEFGH* genes were defined as strains with the PT R-M system. Strains with modification gene clusters but not complete restriction gene clusters were defined as strains with only PT modifications.

### Pangenome analysis.

For S. enterica and K. pneumoniae, all PT R-M strains (425 and 56 strains, respectively) and 450 or 329 randomly selected PT R-M-free strains were chosen for pangenome analysis. For E. coli, 150 PT R-M strains and 202 PT R-M-free strains were randomly selected for the analysis. Bacterial assembly RefSeq files were annotated by Prokka (v1.13) with the parameter “--kingdom Bacteria” (71). Pangenome analysis was performed using Roary v3.13.0 (72) on the generated GFF files. Six threads were used for operation, the multiFASTA alignment of core genes was created using PRANK, and the maximum number of clusters was limited to 70,000.

### Phylogenetic tree.

The core_gene_alignment.aln files obtained by pangenome analysis were used to construct the core gene phylogenetic tree of each bacterium. TrimAl v1.4 (73) was used to remove large gaps and align both ends of the aligned sequences. FastTree v2.1.10 (74) was used to perform this process, using the parameter -nt for the construction of phylogenetic trees based on nucleotide sequences. The confidence level of branches was evaluated with the Shimodaira-Hasegawa test (SH) of the local support value on each node. A value between 0 and 1 and greater than or equal to 0.9 indicates that the corresponding branch is more credible (74). iTOL (https://itol.embl.de) was used to modify the phylogenetic trees, annotating whether the strains on each leaf harbored the PT RM cluster and the total number of AMR genes in the genome.

### Prediction of AMR genes in the genomes.

AMR genes in bacterial assembly RefSeq files were predicted using the Resistance Gene Identifier (RGI) tool (v5.1.0) in the Comprehensive Antimicrobial Resistance Database (CARD) (database v3.0.4) (3). Perfect or strict hits were generated for a genome assembly or genome sequence using default parameters. The tables were generated from precompiled RGI main JSON files, the samples for which were clustered by similarity of resistome and AMR genes organized by AMR gene family.

Differences in the distribution of AMR gene families among different bacteria were drawn in Venn diagrams using the online tool “Calculate and draw custom Venn diagrams” (http://bioinformatics.psb.ugent.be/webtools/Venn/) to perform visual analysis. See Table S3 for the list of AMR gene families. Differences in the distribution of gene numbers of different gene families in each genome were heatmapped using the “heatmap” module in TBtools (v1.089) (75) for visualization. The rows in the heatmap were clustered using the method “complete” to cluster the genomes with a similar distribution of AMR genes together.

For each strain, defining the strain as having any AMR gene in the same gene family meant that the strain had a gene of that gene family, and defining the strain as not having any AMR gene in the same gene family meant that the strain did not have a gene of that gene family. Different strains of the same bacterium were grouped according to the presence of the PT R-M or absence of the PT R-M (PT R-M-free) gene cluster, and the percentage of each AMR gene family in each group in the population was calculated. TBtools, as described above, was used to draw the heatmaps according to the calculation results described above and perform row clustering.

### Prediction of the R-M system and CRISPR-Cas in the genomes.

Methyltransferase and restriction enzymes in bacterial genomes were identified using Restriction-ModificationFinder (https://cge.food.dtu.dk/services/Restriction-ModificationFinder/) (25). Having the same type of methyltransferase and restriction enzyme at the same time is defined as having this type of Me R-M system. CRISPRCasFinder (v4.2.20) was used to identify CRISPR-Cas systems in bacterial genomes, excluding level 1 and level 2 CRISPR array candidates and considering level 3 and level 4 as credible CRISPR arrays (26). Having at least one CRISPR array and one Cas at the same time is considered to have a CRISPR-Cas system in the genome.

### Analysis of upstream and downstream genes of the PT R-M cluster.

According to the position of the PT R-M cluster in the genome, the 5,000-bp sequences upstream (upstream of *dndB*) and downstream (upstream of *dndF*) were extracted. Sequences with a length of up to 5,000 bp were selected from the extracted sequences and annotated with Prokka (version 1.13) with the parameter “--kingdom Bacteria,” and the upstream and downstream gene distribution presence/absence matrices were drawn using the default parameters of Roary v3.13.0. Clustering was performed according to the similarity of gene distribution.

### Prediction of plasmids and prophage sequences in the genomes.

Plasmid replicons in bacterial genomes were analyzed using the CGE Web tool PlasmidFinder v2.1 (https://cge.food.dtu.dk/services/PlasmidFinder/) (76, 77) to screen for potential plasmid sequences. Prediction was performed using the *Enterobacteriales* database. The selection threshold for minimum percent identity was 95%, and the selection minimum percent coverage was 60%. The contigs with plasmid replicons were compared with the sequence lengths of the corresponding reference plasmids given in the prediction results. Plasmid-derived sequences were defined as contigs whose length did not exceed the normal length of the plasmids. According to the screened contigs list, the AMR gene prediction results located on these contigs were extracted from the RGI prediction results of each genome. These AMR genes are considered plasmid-derived AMR genes.

The strain genomes annotated with Prokka with the parameter “--kingdom Bacteria” were used to predict prophage sequences using PhiSpy (v4.2.21) with default parameters (36). The obtained sequences used RGI to predict potential AMR genes.

### Prediction of integrating MGEs.

Integrating MGEs (iMGEs) in bacterial genomes were predicted using MobileElementFinder (software version v1.0.3, database version v1.0.2) (78) with default parameters. For the obtained prediction results, iMGE sequences were extracted from the corresponding genome according to the obtained sequence start and end positions using the “fasta extract” module in TBtools (v1.089) (75), and AMR prediction was performed using RGI to obtain AMR-related iMGE information.

### Unique gene diversity calculations.

The unique gene diversity was calculated based on the matrix of gene presence or absence obtained from pangenome analysis as described previously (13). For each bacterium, any two genomes in the groups with PT R-M or without PT R-M were paired. The genes shared by the two genomes were defined as the core genes, and the sum of all genes in the two genomes was defined as the total genes. The ratio of unique genes to the corresponding total genes is the ratio of unique genes.

### Coevolutionary analysis between PT clusters and the core genes.

The core gene sequences corresponding to strains with PT R-M were extracted from the core_gene_alignment.aln file obtained by pangenome analysis; the *dndBCD* nucleotide sequences in the genomes of these strains were collected according to the BLAST results above using TBtools as described. The core gene sequences or *dnd* cluster sequences were aligned using the default parameters of mafft v7.475 (79). TrimAl v1.4 (73) was used to remove large gaps and align both ends of the aligned sequences. FastTree v2.1.10 (74), as described above, was used to construct the phylogenetic trees based on the obtained alignments of nucleotides.

Dendroscope v3.7.5 (80) was used to perform tanglegram analysis between the phylogenetic trees of *dnd* clusters and the corresponding core genes to visualize the difference between them. ETE3 v3.1.2 (81) was used to evaluate topological similarity between the phylogenetic trees by computing the normalized Robinson-Foulds (nRF) distance. The final value is between 0 and 1. A lower value means a more similar topology between the two phylogenetic trees being compared (82).

### Covariance calculation.

Covariance was calculated using the following formula: $\text{Cov}\left(X,Y\right)=\frac{\sum^{\;}(X_{i}-X)(Y_{j}-Y)}{n}$. *X* indicates the value of AMR genes or iMGE. When the PT R-M system exists, *Y* takes the value 1; otherwise, *Y* takes the value 0. *N* represents the number of samples. The value of covariance reflects the changing trend of two-dimensional random variables. If the covariance is positive, the change trend is consistent; otherwise, the changing trend is the opposite.
