# Supplementary material for: The DNA Phosphorothioation Restriction-Modification System Influences the Antimicrobial Resistance of Pathogenic Bacteria
Source: Microbiol Spectr. 2023 Jan 4;11(1):e03509-22. doi: 10.1128/spectrum.03509-22 (PMC9927239; doi:10.1128/spectrum.03509-22)

Fig. S1 The presence/absence matrix of the upstream and downstream gene distributions of the PT R-M gene cluster in the genome. The rows in the matrix represent strain genomes, the columns represent the genes near the *dnd* cluster, the color of the blocks represents the presence/absence of genes, red means presence, grey means absence. Clustering was performed based on similarity of gene distributions. Genes marked in yellow at the bottom of the matrix mean more frequent occurrences in the group.

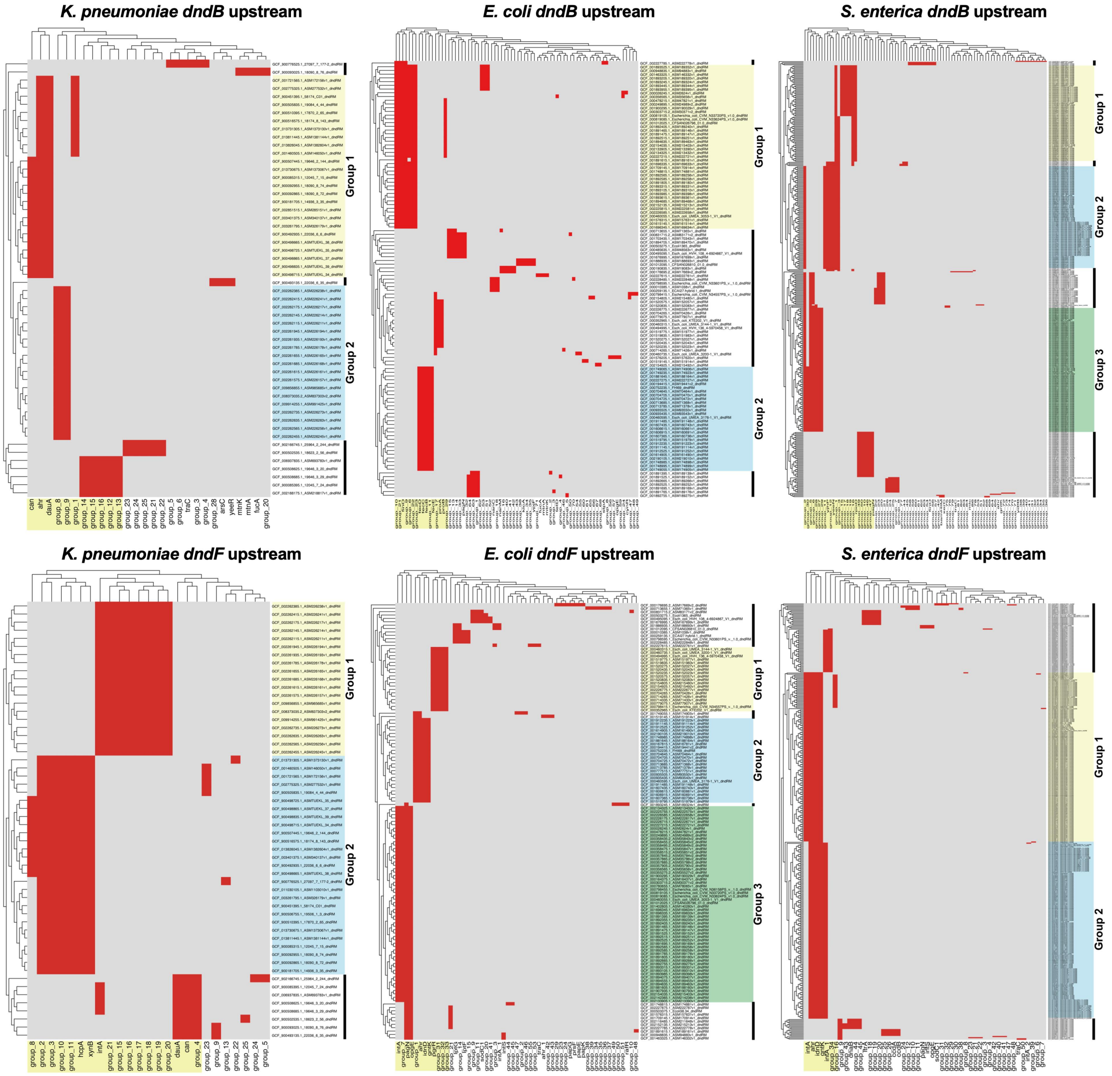

Supplement: Supplemental file 1 — Fig. S1. Download spectrum.03509-22-s0001.pdf, PDF file, 1.4 MB [file spectrum.03509-22-s0001.pdf]
